# The complete chloroplast genome of *Eurya rubiginosa* var. *attenuata* H. T. Chang (Pentaphylacaceae)

**DOI:** 10.1080/23802359.2023.2220433

**Published:** 2023-06-09

**Authors:** Yingshuo Li, Minghao Sun, Yueqi Sun, Mingqiang Wang, Fuwei Zhao

**Affiliations:** a Nanjing Institute of Environmental Sciences, Ministry of Ecology and Environment, Nanjing, China; b Forestry College, Nanjing Forestry University, Nanjing, China; c Academy of Environmental Planning & Design Co., Ltd., Nanjing University, China

**Keywords:** *Eurya rubiginosa* var. *attenuata*, chloroplast genome, phylogenetic analysis, Pentaphylacaceae

## Abstract

*Eurya rubiginosa* var. *attenuata* is a valuable multiuse tree with a long history of use in China. It has great economic and ecological importance and is used for landscape and urban planting, soil improvement, and raw materials for food production. However, genomic studies of *E. rubiginosa* var. *attenuata* are limited. Meanwhile, the classification of this taxon is controversial. In this study, the complete plastome of *E. rubiginosa* var. *attenuata* was successfully sequenced and assembled. The chloroplast genome is 157,215 bp in length with a 37.3% GC content. The chloroplast genome structure includes a quadripartite structure comprising a pair of inverted repeat (IR) sequences of 25,872 bp, a small single-copy (SSC) region of 18,216 bp, and a large single-copy (LSC) region of 87,255 bp. The genome contains 128 genes, including 83 protein-coding genes, 37 tRNA genes, and 8 rRNA genes. Phylogenetic inference based on complete plastome analysis showed that *E. rubiginosa* var. *attenuata* is closely related to *E. alata* and belongs to the family Pentaphylacaceae, which differs from the results of the traditional Engler system. The chloroplast genome sequence assembly and phylogenetic analysis enrich the genetic resources of Pentaphylacaceae and provide a molecular basis for further studies on the phylogeny of the family.

## Introduction

*Eurya rubiginosa* var. *attenuata* H. T. Chang 1954 is an evergreen shrub tree in southern China (Li and Ye 2009). The species is a valuable multi-use tree that can be applied in landscape and urban planting and soil improvement and provides raw materials for food production (Li and Ye 2009). In China, the ash left over from the combustion of *E. rubiginosa* var. *attenuata* has a long history. It is used to make a traditional cake known as Huangguo, which can be eaten during the Spring Festival in southern China. The Flora of China (http://www.iplant.cn/frps) places *E. rubiginosa* var. *attenuata* in the family Pentaphylacaceae, whereas the Engler classification treated it as a member of the family Theaceae (http://www.cfh.ac.cn/). The classification of *E. rubiginosa* var. *attenuata* is controversial. From the chloroplast genome sequence, the systematic position of *E. rubiginosa* var. *attenuata* can be explored. To date, the chloroplast genome of *E. rubiginosa* var. *attenuata* has not been analyzed. In this study, we report the complete genome sequence of *E. rubiginosa* var. *attenuata* and elucidate its phylogenetic relationships.

**Figure 1.**
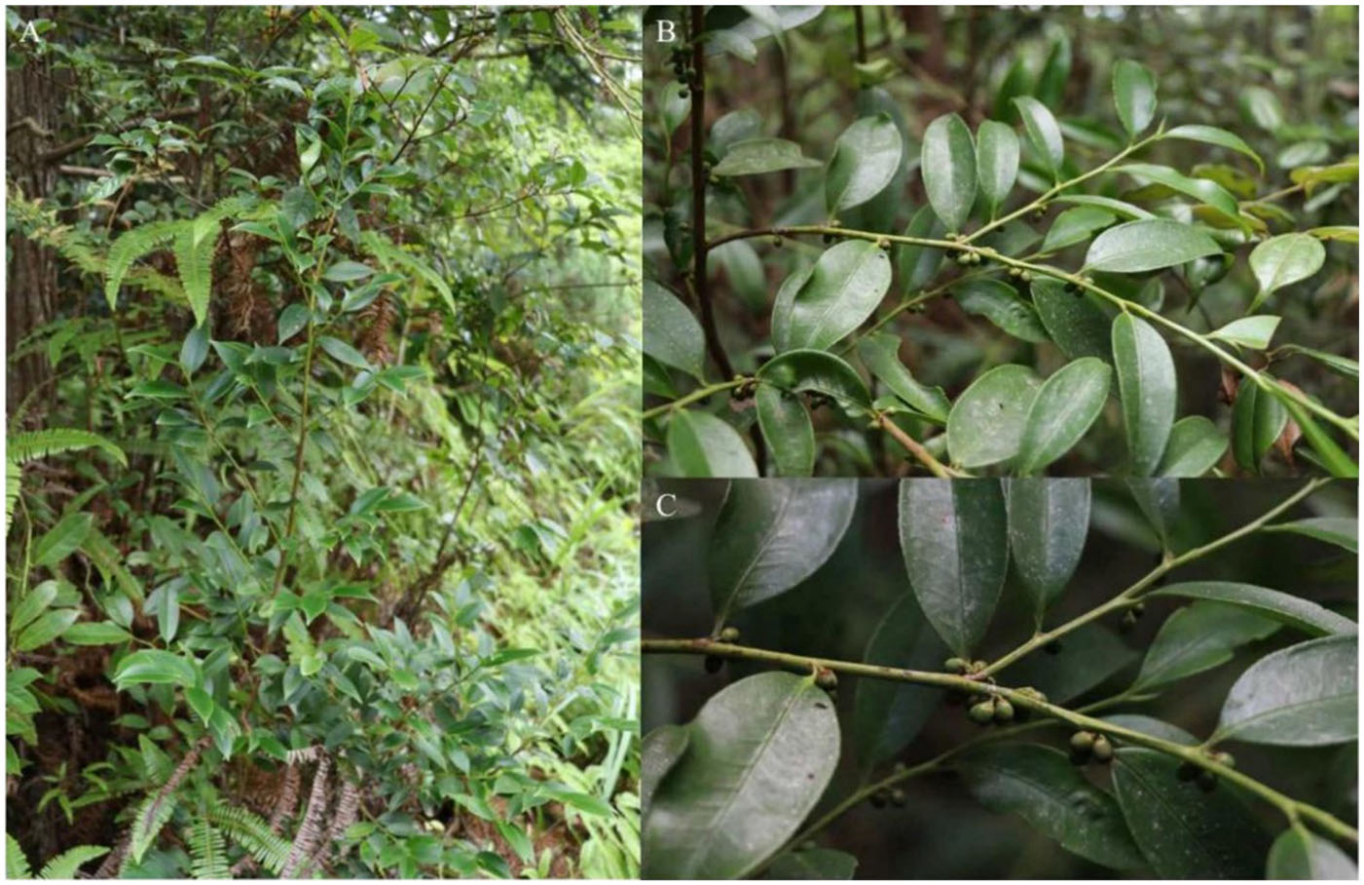
The morphological characteristics of *Eurya rubiginosa* var. *attenuata*. A, B, and C show photos of the whole plant, leaves, and fruits, respectively (photos taken by Yingshuo Li in Qingyuan County, Zhejiang Province, China)

## Materials and methods

The sample of *E. rubiginosa* var*. attenuata* was collected from Xianliang Town, Qingyuan County, Zhejiang Province, China (119° 14′ 19.60″ E, 27° 39′ 12.05″ N), and the voucher specimen was deposited at the Nanjing Institute of Environmental Sciences, Ministry of Ecology and Environment, with number 331126TKQY0001 (https://www.nies.org; contact person: Yingshuo Li, email: 1219755637@qq.com). Total genomic DNA was extracted from the leaves of *E. rubiginosa* var. *attenuata* and sequenced on an Illumina HiSeq XTen platform (San Diego, CA, USA). The insertion size of the sequencing library was 350 bp, and the sequencing strategy was 2*150 bp paired-end. The genome sequence was assembled by NOVOPlasty v.4.3.1 (Dierckxsens et al. 2017). A total of 66,744 reads were mapped to the complete genome sequence of *E. rubiginosa* var. *attenuata* in Geneious Prime 2022.2.2 (Kearse et al. 2012), yielding a coverage of 69× (Figure S1). The assembled chloroplast genome was annotated with CPGview (Liu et al. 2023) and manually corrected for start and stop codons, as well as for intron/exon boundaries to match gene predictions. The genome sequence of *E. rubiginosa* var. *attenuata* has been deposited in GenBank (accession number: ON729444). A molecular phylogenetic tree was generated by the GTR + GAMMA model in RAxML (Stamatakis 2014) with 1000 bootstrap replicates. GenBank was used to obtain the complete chloroplast sequence of *E. rubiginosa* var. *attenuata* and ten related species. The whole plastome sequences were aligned by MAFFT 7.409 (Katoh and Standley 2013) using default settings.

**Figure 2.**
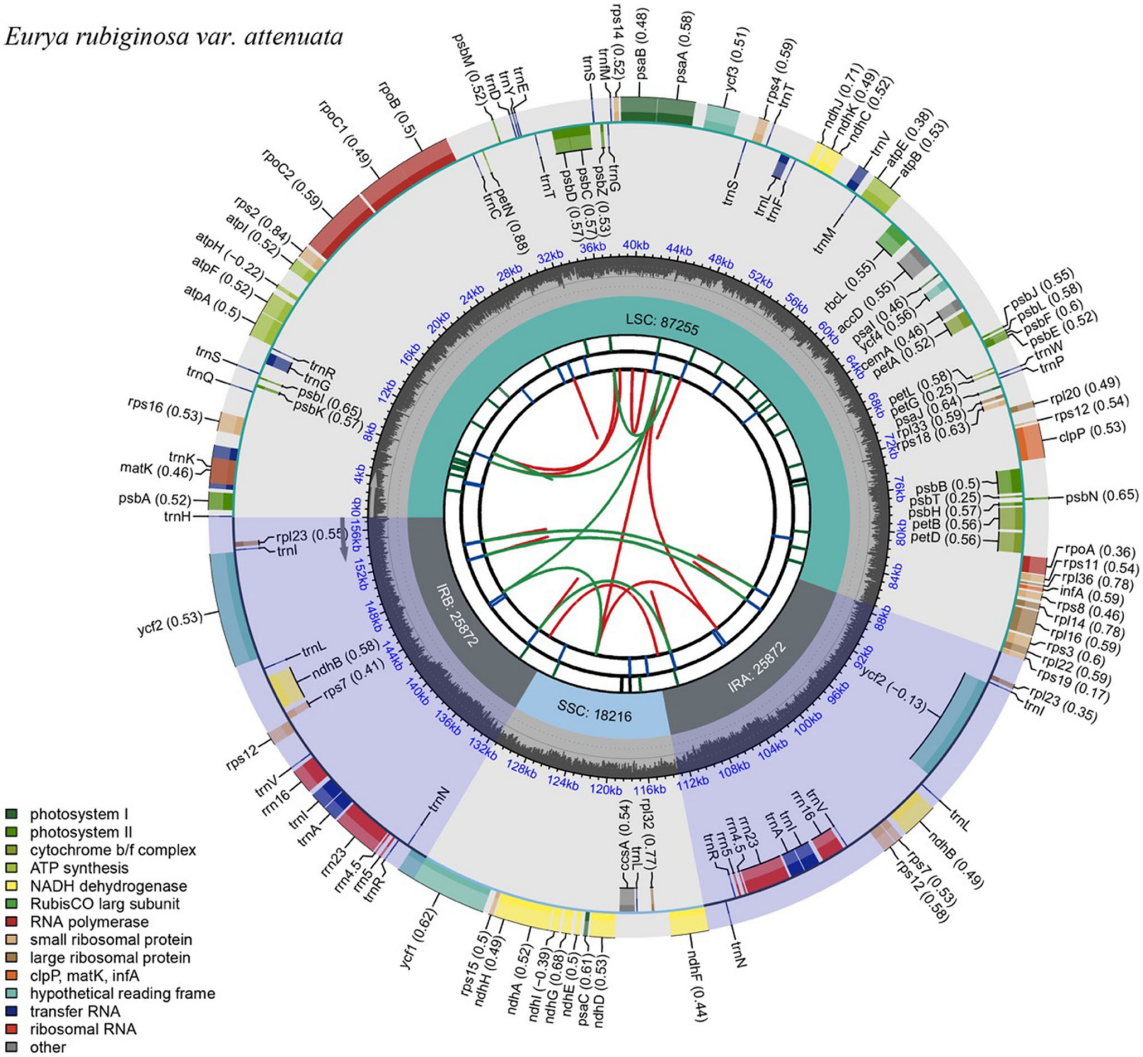
Schematic map of the overall features of the *Eurya rubiginosa* var. *attenuata* chloroplast genome. The circular map of the chloroplast genome was generated using CPGview. The map contains six tracks. From the center going outward, the first circle shows the distributed repeats connected with red (the forward direction) and green (the reverse direction) arcs. The second circle shows the long tandem repeats as short blue bars. The third track shows the microsatellite sequences as short bars with different colors. The fourth circle shows the sizes of the chloroplast genome regions, including the small single-copy (SSC), inverted repeat (IRa and IRb), and large single-copy (LSC) regions. The LSC and SSC regions measure 87255 bp and 18216 bp, respectively, while the inverted repeat regions (IRa and IRb) are both 25872 bp in length. The fifth track shows the GC content along the genome. The sixth circle shows the genes with different colors based on their functional groups. The transcription directions for the inner and outer genes are clockwise and counterclockwise, respectively.

**Figure 3.**
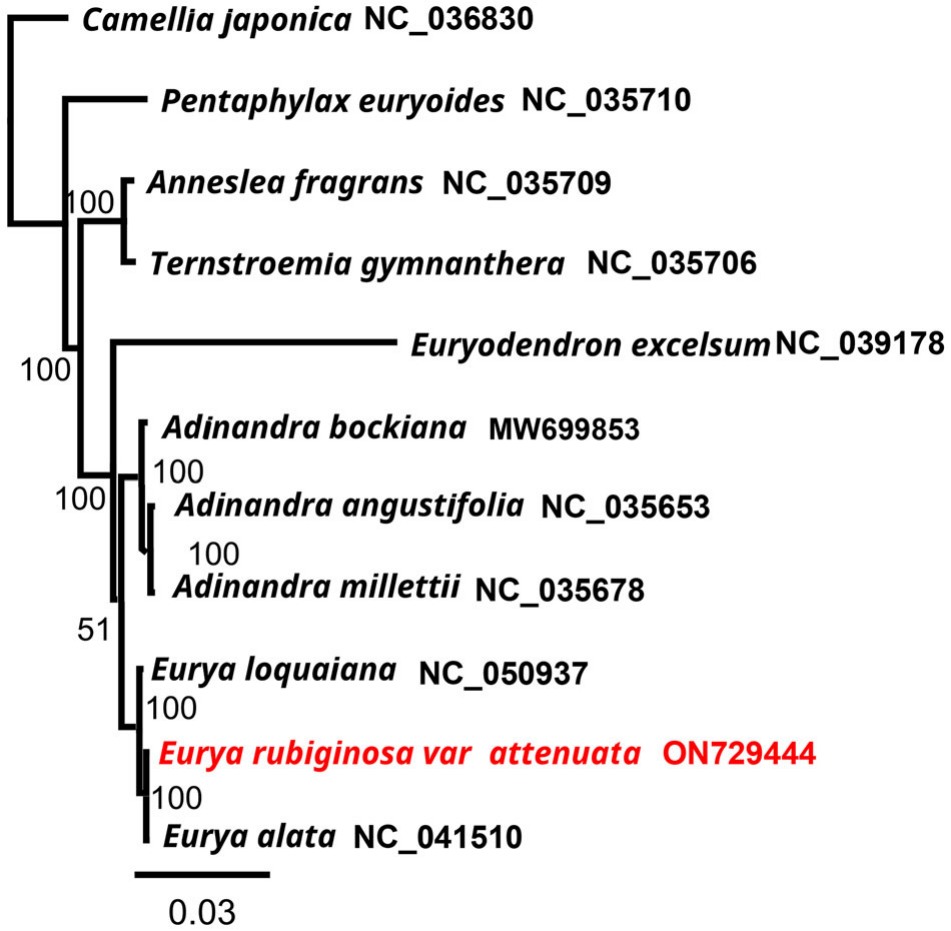
Tree showing the phylogenetic relationships of *Eurya rubiginosa* var. *attenuata*. The numbers on the branches represent the bootstrap values based on 1000 replicates. The sequences used for tree construction are as follows: *Camellia japonica* (NC_036830), *Pentaphylax euryoides* (NC_035710; Yu et al. 2017), *Anneslea fragrans* (NC_035709; Yu et al. 2017), *Ternstroemia gymnanthera* (NC_035706; Yu et al. 2017), *Euryodendron excelsum* (NC_039178; Shi et al. 2019), *Adinandra bockiana* (MW699853), *A. angustifolia* (NC_035653; Yu et al. 2017), *A. millettii* (NC_035678; Yu et al. 2017), *Eurya loquaiana* (NC_050937; Wang et al. 2020), *E. rubiginosa* var. *attenuata* (ON729444), and *E. alata* (NC_041510; Lin et al. 2019).

## Results

The total length of the *E. rubiginosa* var. *attenuata* chloroplast genome is 157,215 bp, of which the inverted repeat (IR) sequence is 25,872 bp and the large single-copy (LSC) and small single copy (SSC) regions are 87,255 bp and 18,216 bp, respectively. The GC content of the complete chloroplast genome is 37.3%, with 43%, 35.3%, and 31% in the IR, LSC and SSC regions, respectively. The genome has 128 genes, including 83 protein-coding genes, 37 tRNA genes and 8 rRNA genes. Among these, 16 genes were repeated in IR regions, including 5 protein-coding genes (*rps12*, *rps7*, *ndhB*, *ycf*2, and rpl23), 7 tRNA genes (*trnN-GUU*, *trnR-ACG*, *trnA-UGC*, *trnI-GAU*, *trnV-GAC*, *trnL-CAA*, and *trnI-CAU*) and 4 rRNA genes (*rrn4.5*, *rrn5*, *rrn16,* and *rrn23*). Nineteen genes contained an intron, including 11 protein-coding genes (*atpF*, *rpoC1*, *rps*12*2, *petB*, *petD*, ndhB*2, *rpl*2, *rpl16*, and *ndhA*) and 8 tRNA genes (*trnK-UUU*, trnI-GAU*2, *trnG-UCC*, *trnL-UAA*, *trnV-UAC*, and trnA-UGC*2), while *ycf*3 and *clp*P possessed two introns. The gene order and the length of the genes are similar to those of *E. alata* (NC_041510) and *E. loquaiana* (NC_050937).

To investigate the phylogenetic placement of *E. rubiginosa* var. *attenuata*, the phylogenetic tree, consisting of the complete cp genomes of *E. rubiginosa* var. *attenuata* and 10 other species of Pentaphylacaceae, was reconstructed using *Camellia japonica* as an outgroup (Figure 3). The phylogenetic tree revealed that *E. rubiginosa* var. *attenuata* was closely related to *E. alata* (NC_041510), and plants in *Eurya* had a close relationship with *Adinandra.* All seven Pentaphylacaceae species formed a monophyletic group.

## Discussion and conclusion

With its highly conserved sequence, the chloroplast genome, as a superbarcode, can provide information for resolving complex evolutionary relationships between species (Li et al. 2020). In this study, the complete plastome of *E. rubiginosa* var. *attenuata* was successfully sequenced and assembled for the first time. The chloroplast genome of *E. rubiginosa* var. *attenuata* shows a quadripartite structure similar to that in the majority of other angiosperms (Liu et al. 2022), consisting of a pair of inverted repeats (IRs), a small single-copy (SSC) region, and a large single-copy (LSC) region. The phylogeny reconstructed from complete plastomes revealed that *E. rubiginosa* var. *attenuata* is closely related to *E. alata*, forming a monophyletic clade with the rest of the Pentaphylacaceae species. The results for *E. rubiginosa* var. *attenuata*, a member of the Pentaphylacaceae family, differed from those reported under the Engler system. Reasons for this discrepancy may include incomplete lineage sorting, species and other taxa with similarly recent divergence times and low levels of genetic variation, hybridization, or taxonomic oversplitting (Yu et al. 2022). Overall, our study provides a molecular basis for further phylogenetic analysis of the family, the exploitation and utilization of plants, and resource protection.

## Supplementary Material

Supplemental Material

Supplemental Material

Supplemental Material

## Data Availability

The genome sequence data that support the findings of this study are openly available in GenBank of NCBI at (https://www.ncbi.nlm.nih.gov/) under accession no. ON729444. The associated BioProject, SRA, and Bio-Sample numbers are PRJNA905216, SRR22406071 and SAMN31867763, respectively.
