# Supplementary material for: The complete chloroplast genome of Eurya rubiginosa var. attenuata H. T. Chang (Pentaphylacaceae)
Source: Mitochondrial DNA B Resour. 2023 Jun 9;8(6):639–42. doi: 10.1080/23802359.2023.2220433 (PMC10259296; doi:10.1080/23802359.2023.2220433)
Supplement: Supplemental Material [file TMDN_A_2220433_SM9574.docx]

Figure captions

Figure S1. The overall coverage depth of *Eurya rubiginosa* var. *attenuata* chloroplast genome assembly.

Figure S2. Schematic map of the cis-splicing genes in the chloroplast genome of *Eurya rubiginosa* var. *attenuata*.

Figure S3. Schematic map of the trans-splicing genes in the chloroplast genome of *Eurya rubiginosa* var. *attenuata*.


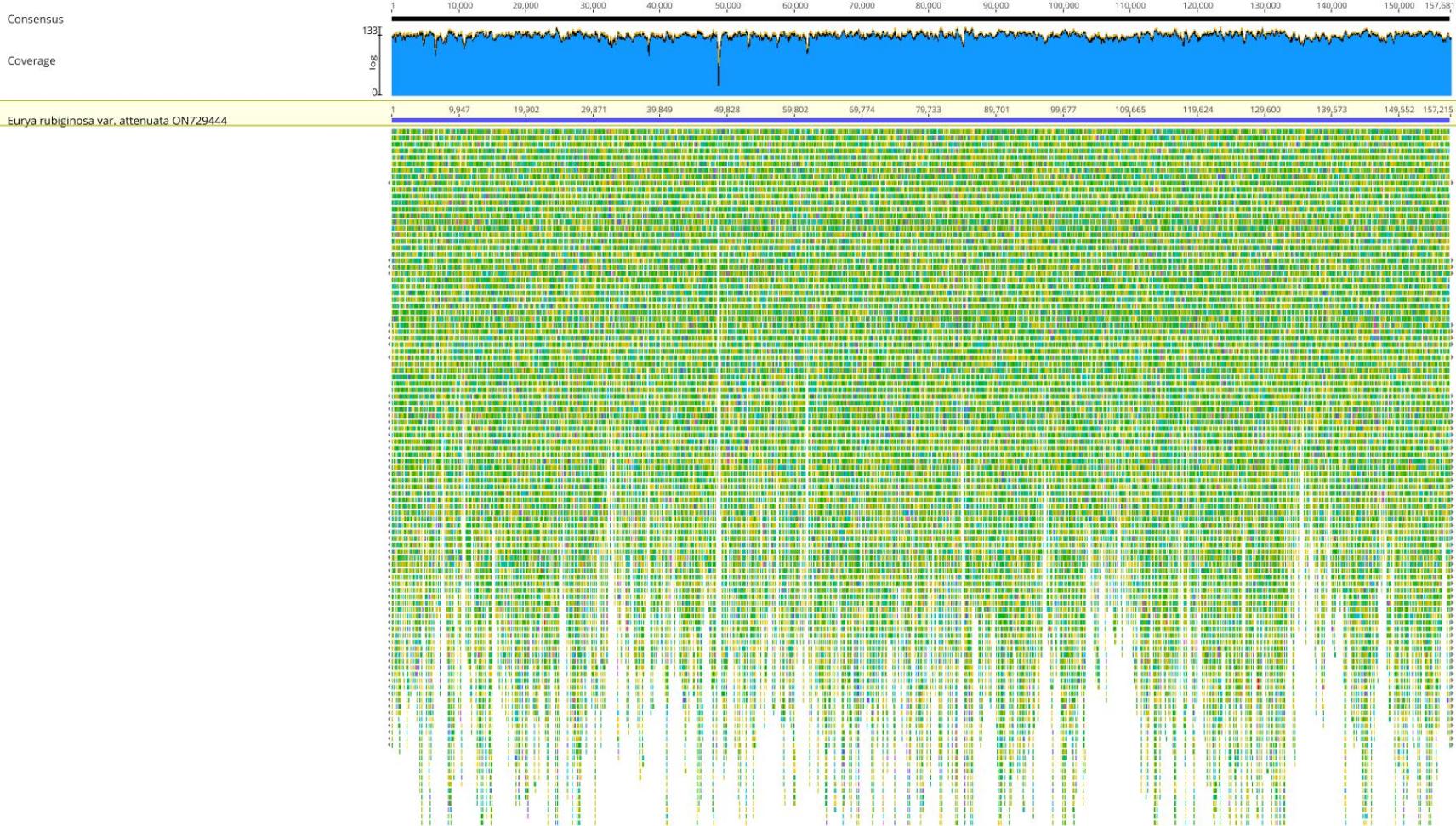


Figure S1. The overall coverage depth of *Eurya rubiginosa* var. *attenuata* chloroplast genome assembly. This image was created by aligning DNA-Seq data to the entire chloroplast genome with Geneious Prime. The number of sequences at each location is indicated by the height of the blue graph. A total of 66,744 reads were mapped to the *E. rubiginosa* var. *attenuata* genome sequence.


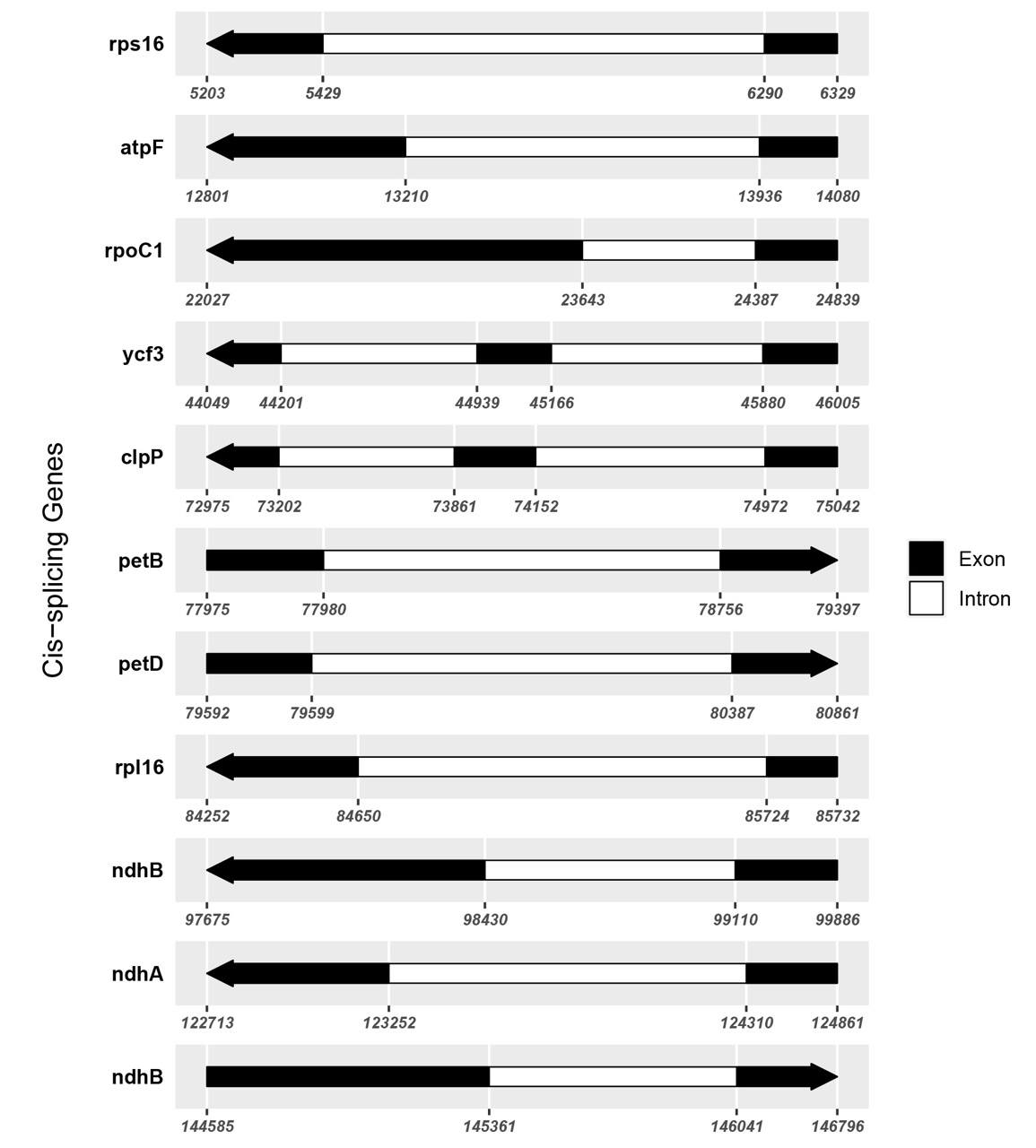


Figure S2. Schematic map of the cis-splicing genes in the chloroplast genome of *Eurya rubiginosa* var. *attenuata*. The map was generated using CPGview. The gene names are shown on the left, and the gene structures are on the right. The exons are shown as black and the introns as white. The arrow indicates the direction of the gene.


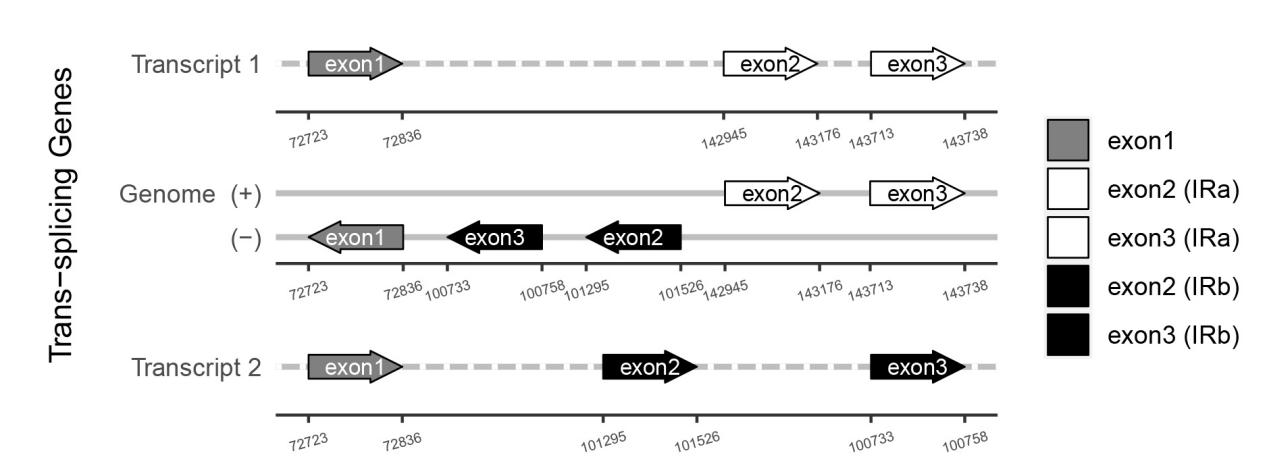


Figure S3. Schematic map of the trans-splicing genes rps12 in the chloroplast genome of *Eurya rubiginosa* var. *attenuata.* The map was generated using CPGview. It has three unique exons. Two exons are duplicated as they are located in the IR regions.
