# Supplementary figures and images for: The complete chloroplast genome of Eurya rubiginosa var. attenuata H. T. Chang (Pentaphylacaceae)
Source: Mitochondrial DNA B Resour. 2023 Jun 9;8(6):639–42. doi: 10.1080/23802359.2023.2220433 (PMC10259296; doi:10.1080/23802359.2023.2220433)

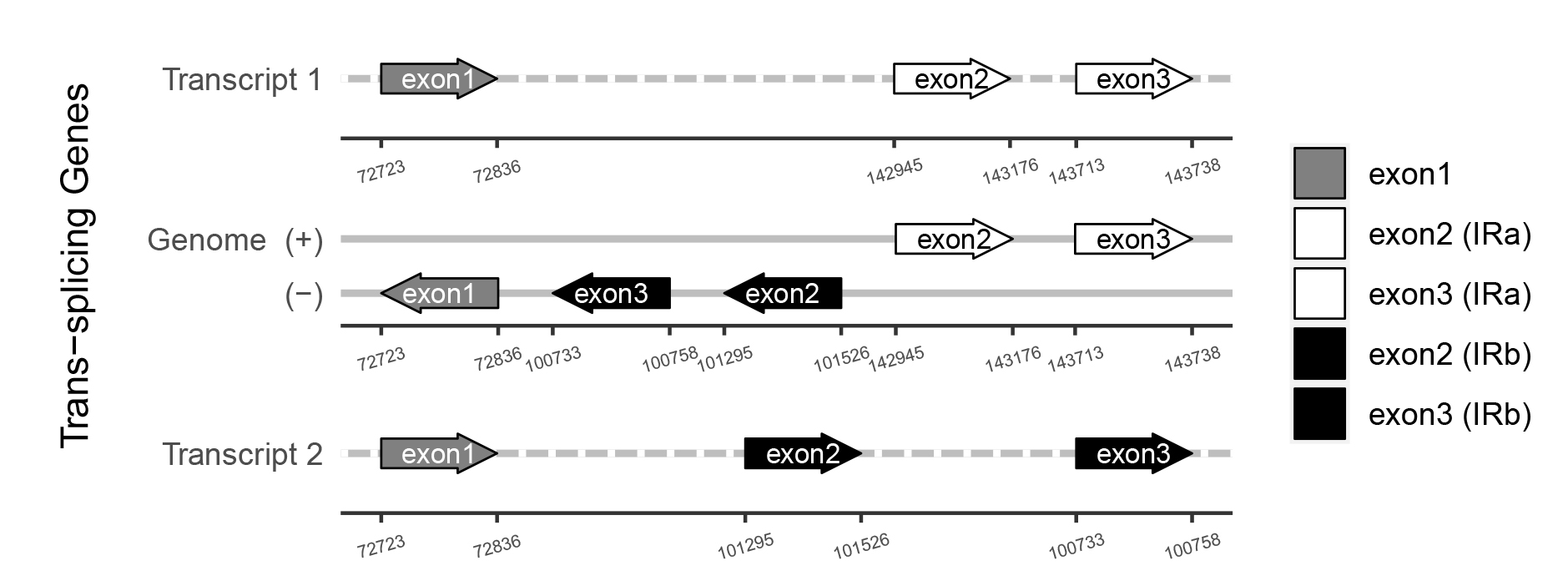

Supplement: Supplemental Material [file TMDN_A_2220433_SM9573.tif]

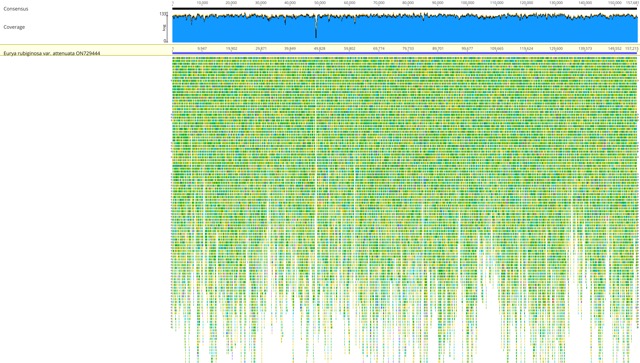

Supplement: Supplemental Material [file TMDN_A_2220433_SM9570.jpeg]
